# Left Posterior Fascicular Pacing

**DOI:** 10.19102/icrm.2021.120506

**Published:** 2021-05-15

**Authors:** Shunmuga Sundaram Ponnusamy, Thabish Syed, Surya Kumar

**Affiliations:** ^1^Department of Cardiology, Velammal Medical College Hospital and Research Institute, Madurai, Tamil Nadu, India

**Keywords:** Left bundle pacing, posterior fascicle, peak left ventricular activation time

## Abstract

Left bundle branch pacing (LBBP) is emerging as an alternative to His bundle pacing that overcomes the latter’s limitations. Several studies have reported on the safety, efficacy, and electrophysiological properties of LBBP, while postoperative success rates range from 80.5% to 94%. The left posterior fascicle is composed of broad bands of fibers coursing inferiorly and posteriorly toward the papillary muscle, while the anterior fascicle is a thin, tendon-like structure. We report a case of a 70-year-old man in whom left posterior fascicular pacing was done after LBBP failed. We were able to demonstrate all the features of left posterior fascicular capture, including fascicular potential and a left anterior hemiblock pattern, using surface 12-lead electrocardiography. Left posterior fascicular pacing could be an alternative technique when attempts to deploy LBBP fail.

## Introduction

His bundle pacing (HBP) has emerged as an excellent alternative to right ventricular (RV) apical pacing wherein the cardiac conduction system is captured directly. However, although the use of HBP avoids RV pacing–related complications, its use is also limited by technical challenges, high capture threshold, lead dislodgement, and the need for redo procedures.^1^ Huang et al. reported an alternative strategy to HBP that involves direct capture of the LBB.^2^ LBB pacing (LBBP) provides a low and constant threshold with excellent lead stability and reported success rates that vary between 80.5% and 94%.^3,4^ In this report, we describe the case of a 70-year-old man with symptomatic sinus node dysfunction for whom left posterior fascicular pacing was performed after a failure to deploy LBBP.

## Case presentation

**Figure 1:**
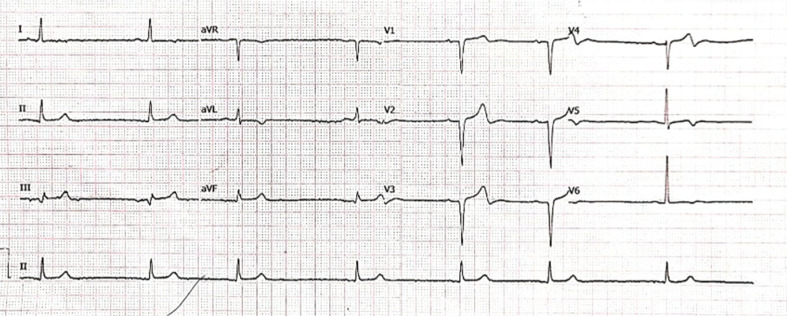
Baseline 12-lead ECG showing sinus bradycardia with normal QRS duration.

A 70-year-old male with symptomatic sinus node dysfunction and normal coronaries **(Figure 1)** presented to us for permanent pacemaker implantation. He had recurrent episodes of unprovoked syncope. Echocardiography showed normal left ventricular function and 24-hour Holter monitoring recorded multiple sinus pauses of greater than three seconds.

**Figure 2:**
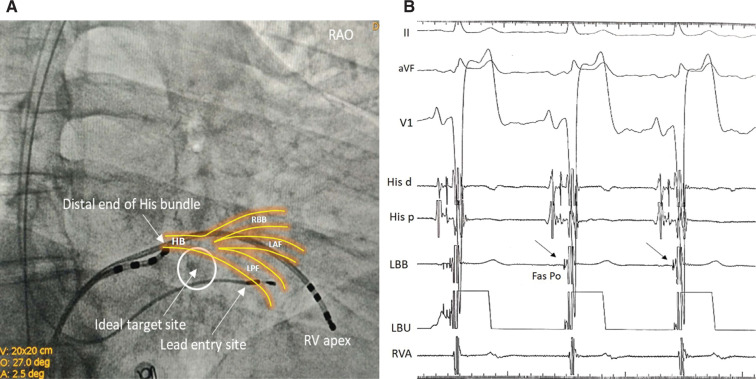
Left posterior fascicular pacing. **A:** A right anterior oblique 30° fluoroscopic view showing the ideal target site (white circled area) and the actual placement of the lead to capture the posterior fascicle. RV: right ventricle. **B:** Intracardiac pacing lead EGMs recorded during sinus rhythm showing a sharp fascicular potential (black arrow) preceding the local ventricular EGM. LBB: left bundle branch pacing lead EGM; His d and His p: His bundle EGM distal and proximal; RAO: right anterior oblique; RVA: right ventricular activation distal EGM; Fas Po: fascicular potential; RBB: right bundle branch; LAF: left anterior fascicle: LPF: left posterior fascicle.

**Figure 3:**
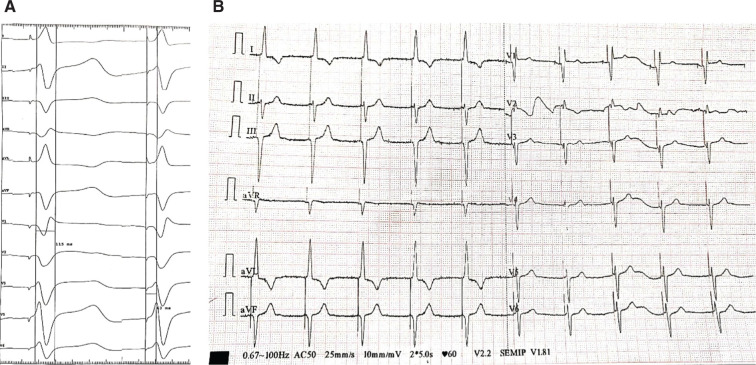
**A:** The peak left ventricular activation time as measured from lead V5 was 63 ms and the paced QRS duration was 115 ms. **B:** A 12-lead ECG showing qR in lead V1, rS in inferior leads, and a deep S-wave in lead V6 suggestive of left posterior fascicular capture manifesting as left anterior hemiblock.

After obtaining informed consent from the patient for the procedure, LBBP was attempted. Continuous recording of a 12-lead electrocardiogram (ECG) and intracardiac electrograms (EGMs) were recorded using an electrophysiology (EP) system (Abbott, Chicago, IL, USA). As atrial pacing showed atrioventricular Wenchebacking at 110 bpm, a dual-chamber pacemaker was considered. A 4.1-French lumenless 3830 SelectSecure™ lead and a C315 sheath (Medtronic, Minneapolis, MN, USA) were used to capture the left bundle. An attempt to deploy this at a site 1.5 cm below the His bundle along the imaginary line joining the distal His bundle signal to the RV apex failed. A sheath-in-sheath technique using a coronary sinus sheath to provide additional support was also tried without success. Subsequently, a C315 sheath was positioned 2 cm inferior to the previously attempted site toward the RV apex **(Figure 2A)**. From here, the pacing lead could be positioned deep inside the septum using five rapid turns. The lead-tip EGM showed a sharp fascicular potential preceding the local ventricular signal **(Figure 2B)** with the potential to surface QRS duration of 18 ms. Unipolar pacing showed qR in lead V1, a peak left ventricular activation time of 63 ms in lead V5, and a QRS duration of 115 ms **(Figure 3A)**. Finally, a 12-lead ECG showed rS in inferior leads and a deep S-wave in lead V6 suggestive for left anterior fascicular conduction delay.

Together, the abovementioned features suggested successful capture of the left posterior fascicle by the pacing lead, resulting in a sharp fascicular potential preceding the local ventricular EGM during sinus rhythm and a left anterior hemiblock pattern in the surface ECG. The unipolar pacing impedance was 680 Ω, and the capture threshold was 0.5 V at a 0.6-ms pulse width. The sensed R-wave was 10 mV. The atrial lead was positioned at the right atrial appendage. The final paced ECG showed qR in lead V1 and a left anterior hemiblock pattern with a QRS duration of 115 ms **(Figure 3B)**. The patient recovered well and was discharged the next day without any complications. Three months later, the pacing parameters remained stable with the pacing threshold at 0.5 V at a 0.6-ms pulse width and a sensed R-wave of 9.5 mV. No episodes of syncope occurred following pacemaker implantation.

It is believed that the difficulty encountered in this case was likely due to an inappropriate sheath–septum orientation and the inability of the lead to penetrate sufficiently deep enough into the septum. In such cases, left posterior fascicular pacing can be attempted to capture the conduction system.

## Discussion

Huang et al. first described direct capture of the LBB by deep septal pacing.^2^ Since then, many studies have suggested the feasibility, safety, and electrophysiological properties of left bundle pacing for the management of symptomatic bradyarrhythmias.^4^ The left bundle separates from the branching portion of the His bundle underneath the membranous septum. It gives rise to two branches, the anterior and posterior fascicles, each heading toward the corresponding papillary muscle.^5^ The left posterior fascicle is composed of multiple fibers distributed over a broad area in contrast with the anterior fascicle, which is essentially a thin tendon.

Li et al.^3^ reported a success rate of 80.5% for LBBP in patients with bradycardia. These authors provided several explanations for the possible failure of left bundle capture, including (1) the inability of the lead to penetrate sufficiently deep enough into the septum; (2) existence of a malpositioned sheath so that the lead cannot go perpendicularly; and (3) a failure of the lead tip to advance due to local hypertrophy. In patients with ischemic cardiomyopathy, a septal scar is an additional factor to consider in unsuccessful positioning of the LBBP lead.

If the left bundle cannot be captured, then posterior fascicular pacing can be attempted by placing the lead 2 cm inferiorly toward the RV apex. This will increase the success rate of conduction system capture. In patients without infra-Hisian complete heart block and complete LBBB, sharp fascicular potentials can be demonstrated preceding the local ventricular EGM during sinus rhythm, as shown in our case. The interval between the fascicular potential to the surface QRS will be less than 20 ms. The paced QRS will have a short LVAT (63 ms in our case) and a left anterior hemiblock pattern.

## Conclusion

Although LBBP has a better learning curve than HBP, certain factors limit the capacity to achieve successful lead placement with the latter. In patients in whom the LBB cannot be captured, left posterior fascicular pacing can be attempted before opting for alternative strategies. With further innovations in tools and techniques, the success rate and efficacy of conduction system pacing are expected to increase.

## References

[1] Zanon F, Ellenbogen KA, Dandamudi G (2018). Permanent His-bundle pacing: a systematic literature review and meta-analysis. Europace.

[2] Huang W, Su L, Wu S (2017). A novel pacing strategy with low and stable output: pacing the left bundle branch immediately beyond the conduction block. Can J Cardiol.

[3] Li Y, Chen K, Dai Y (2019). Left bundle branch pacing for symptomatic bradycardia: implant success rate, safety and pacing characteristics. Heart Rhythm.

[4] Ponnusamy SS, Arora V, Namboodiri N (2020). Left bundle branch pacing: a comprehensive review. J Cardiovasc Electrophysiol.

[5] Rosenbaum MB, Elizari MV, Lazzari JO (1970). The Hemiblocks: New Concepts of Intraventricular Conduction based on Human, Anatomical and Clinical Studies.

